# “How” and “what” matters: Sampling method affects biodiversity estimates of reef fishes

**DOI:** 10.1002/ece3.2979

**Published:** 2017-05-30

**Authors:** Néstor E. Bosch, Jorge M. S. Gonçalves, Karim Erzini, Fernando Tuya

**Affiliations:** ^1^ Grupo en Biodiversidad y ConservaciónIU‐ECOAQUAUniversidad de Las Palmas de Gran CanariaLas Palmas de G.C.Canary IslandsSpain; ^2^ Centro de Ciências do Mar (CCMAR)Universidade do AlgarveFaroPortugal

**Keywords:** asymptotic richness, baited remote underwater video, biodiversity patterns, cost‐efficiency, fish traps, underwater visual census

## Abstract

Understanding changes in biodiversity requires the implementation of monitoring programs encompassing different dimensions of biodiversity through varying sampling techniques. In this work, fish assemblages associated with the “outer” and “inner” sides of four marinas, two at the Canary Islands and two at southern Portugal, were investigated using three complementary sampling techniques: underwater visual censuses (UVCs), baited cameras (BCs), and fish traps (FTs). We firstly investigated the complementarity of these sampling methods to describe species composition. Then, we investigated differences in taxonomic (TD), phylogenetic (PD) and functional diversity (FD) between sides of the marinas according to each sampling method. Finally, we explored the applicability/reproducibility of each sampling technique to characterize fish assemblages according to these metrics of diversity. UVCs and BCs provided complementary information, in terms of the number and abundances of species, while FTs sampled a particular assemblage. Patterns of TD, PD, and FD between sides of the marinas varied depending on the sampling method. UVC was the most cost‐efficient technique, in terms of personnel hours, and it is recommended for local studies. However, for large‐scale studies, BCs are recommended, as it covers greater spatio‐temporal scales by a lower cost. Our study highlights the need to implement complementary sampling techniques to monitor ecological change, at various dimensions of biodiversity. The results presented here will be useful for optimizing future monitoring programs.

## 1 Introduction

The extent to which anthropogenic activities erode biodiversity and underlying ecosystems services is a central topic in current conservation (Cardinale et al., 2012; Chapin et al., 2000; Naeem, Duffy, & Zavaleta, 2012). Contemporary marine management approaches aiming at protecting ecological functions of natural communities, rather than the species per se, requires a better understanding of which aspects (“dimensions”) of biodiversity (e.g., taxonomic diversity, phylogenetic diversity, genetic diversity, functional diversity, and landscape diversity) are ecologically relevant to sustain ecosystem processes and functions (Cadotte, Dinnage, & Tilman, 2012; Díaz & Cabido, 2001; Tilman et al., 1997).

In the past decades, most studies focusing on biodiversity have used conventional diversity metrics, which are based on the number of taxonomically distinct entities and their abundances (i.e., taxonomic diversity, hereinafter TD) (Gaston, 2000; Worm et al., 2006). However, ecosystem functions are mediated by the functional characteristics (i.e., ecological traits) of organisms rather than by their taxonomic identity (Cadotte, 2011); hence, not all the species contribute equally to ecosystem processes (Luck et al., 2009). In this context, phylogenetic diversity metrics (hereinafter PD) and functional diversity metrics (hereinafter FD) have been increasingly used in conservation and ecology. Phylogenetic diversity metrics measure the “relatedness” of species in a community based on their evolutionary history (Cadotte et al., 2010), while FD metrics measure the similarity among species from their functional attributes (e.g., morphological, physiological, reproductive, or behavioral) (Petchey & Gaston, 2002).

The growing need to describe changes in biodiversity through space and time requires the implementation of monitoring programs at local and regional scales. Understanding how different survey methods perform and how varying sampling methods affect biodiversity estimates is essential. Several methods exist to monitor fishes in shallow water marine environments (Murphy & Jenkins, 2010), including underwater visual census techniques (hereinafter UVC), baited cameras (hereinafter BC), and various fishing techniques, for example, fish traps (hereinafter FT). Each method has advantages and limitations that have been thoroughly explored (Edgar, Barrett, & Morton, 2004; Mallet & Pelletier, 2014; Thompson & Mapstone, 1997). Although extrinsic sources of error beyond the method itself (e.g., interobserver variability) can be minimized by standardized protocols and robust sampling designs, methodological bias is largely inevitable, particularly when evaluating multispecies fish assemblages (MacNeil et al., 2008). For instance, BCs have proved to be effective at recording large mobile predatory fish species, which usually avoid divers (Langlois et al., 2010; Willis & Babcock, 2000; Willis, Millar, & Babcock, 2000), while UVCs have proved to be more useful in recording cryptic and herbivorous species (Colton & Swearer, 2010; Lowry, Folpp, Gregson, & Suthers, 2012; Stobart et al., 2007). In turn, different sampling methods can yield different estimates of population mean and variance (Andrew & Mapstone, 1987), varying the statistical power to detect a change in whatever variable of interest (Winer, 1991). This might have severe consequences in environmental management, as we increase the probability of committing a type II error (i.e., the probability of retaining the null hypothesis, when it is false), and might result in misleading conclusions. Ideally, a sampling technique that maximizes accuracy and precision with a minimum cost should maximize the efficiency and reliability of monitoring programs (Underwood, 1981).

Although several authors have suggested that multiple methods should be used concurrently, to encompass the full range of species inhabiting a local area (Baker et al., 2016; Watson, Harvey, Anderson, & Kendrick, 2005), the majority of studies are based on surveys conducted with a single method. Several authors have compared UVC and BC (Colton & Swearer, 2010; Langlois et al., 2010; Stobart et al., 2007), and, to a lesser extent, UVC or BC with traditional extractive sampling techniques, such as FT (Harvey et al., 2012). However, in all of these studies, comparisons were based on the number of species and their relative abundance (i.e., TD). Thus, the extent to which the use of different sampling techniques to describe fish assemblages may have an effect on FD and PD metrics is unknown and represents an important step in diversity research (Robinson et al., 2014). Furthermore, studies focusing on patterns of TD, PD, and FD of fishes are still scant, particularly at local scales (Micheli & Halpern, 2005; Stuart‐Smith et al., 2013; Villéger, Miranda, Hernández, & Mouillot, 2010).

In this work, we studied fish assemblages at the “inner” and “outer” sides of four marinas, two at Gran Canaria Island (Canary Islands) and two at southern Portugal (Algarve coast). Differences in the composition and abundance of fish assemblages were investigated using three complementary sampling techniques (UVC, BC, and FT); this provided various diversity metrics through varying sampling methods. We used this case study to address: (1) the degree of similarity in the composition and abundance of fish assemblages between the three sampling methods, and (2) differences in taxonomic, phylogenetic, and functional diversity between the “inner” and “outer” sides of marinas. A complete evaluation of the suitability of different sampling methods to describe spatial and temporal community patterns requires consideration on their costs and reproducibility/applicability (Langlois et al., 2010; Watson et al., 2005). The time to conduct a single sample of each method was calculated to produce a standardized metric, what allowed us to test: (3) the adequacy of the sampling methods to characterize fish communities, (4) the power of each method to detect significant changes in taxonomic, phylogenetic, and functional diversity, and (5) the cost‐efficiency of the sampling methods. This information is crucial to develop monitoring programs taking advantage of the resources and time available.

## 2 Materials and Methods

### 2.1 Study area

**Figure 1:**
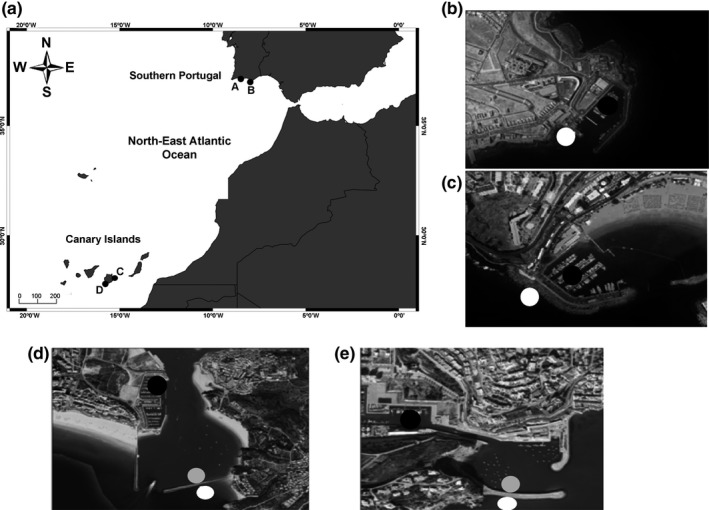
Location of marinas in southern Portugal (Portimão, a; and Albufeira, b) and Gran Canaria Island (Taliarte, c; and Puerto Rico, d), including the “inner” (black symbols), “middle” (gray symbols), and “outer” sides of marinas (white symbols)

This study was carried out at two different ecoregions (i.e., areas of relatively homogenous species composition) within the Lusitanian province in the temperate northeastern Atlantic Ocean realm (Spalding et al., 2007): Gran Canaria Island (Canary Islands) and southern Portugal (Algarve coast). In each ecoregion, we selected two marinas of similar size (<0.1 km^2^): Albufeira (0.083 km^2^; 37°05′02.90″N, 8°16′03.55″W) and Portimão (0.063 km^2^; 37°08′03.00″N, 8°31′49.98″W) in southern Portugal; Taliarte (0.030 km^2^; 27°59′25.74″N, 15°22′05.37″W) and Puerto Rico (0.014 km^2^; 27°59′25.74″N, 15°22′05.37″W) in Gran Canaria Island (Figure 1a). At each marina from Gran Canaria Island, we sampled at two sides, corresponding to the “inner” (inside) and the “outer” (“open ocean”) sides of each marina (Figure 1b,c). At southern Portugal, however, the “inner” and “outer” (“open ocean”) sides are separated by a channel, in the case of Albufeira, and by an estuary in the case of Portimão (Figure 1d,e). Therefore, three different sides with respect to distance from the open ocean were established: “inner,” “middle,” and “outer.” For practical reasons, only one sector of the marinas in Albufeira and Portimão was selected (see details in Figure 1d,e). Despite the specific spatial configuration of each marina, all of them are composed of floating pontoons, small boulders, sand and/or mud banks in the “inner” parts, and big concrete blocks interspersed with sandy patches in the “outer” parts. Although tens of m separate “inner” and “outer” sides, there exist clear artificial boundaries.

### 2.2 Data collection and analyses

Fish assemblages were evaluated, during summer of 2015 in Gran Canaria Island, and in winter of 2016 in the Algarve coast, using three complementary sampling techniques: UVC, FT, and BC. All samples were taken at a depth range between 3 and 5 m and during daylight hours (i.e., 11:00 and 18:00 hours at Gran Canaria Island, 10:00 and 17:00 hours at southern Portugal). At the “inner” sides, all samples were randomly distributed within the area of the marinas; at the “outer” sides, samples were randomly distributed across the seawalls, which vary in length: 100 m at Taliarte, 347 m at Puerto Rico, 321 m at Albufeira, and 610 m at Portimão. The “inner,” “middle,” and “outer” sides of each marina were generally sampled in the same day, and the two marinas within each ecoregion were sampled within a period of 1–4 weeks. The three methods were carried out over three consecutive days to reduce temporal variability (Birt, Harvey, & Langlois, 2012). As both regions are geographically distinct, and were sampled at different seasons and with different bait types, we analyzed both data series independently.

#### 2.2.1 Underwater visual censuses

At each sampling side within each marina, *n* = 6, 10 m length × 4 m wide (40 m^2^), transects, separated by at least 10 m, were deployed by the same SCUBA diver. Along each transect, the diver annotated the abundances of each fish species, on waterproof paper, according to standard procedures for the study region and elsewhere (Tuya, Boyra, Sanchez‐Jerez, & Haroun, 2005; Tuya, Wernberg, & Thomsen, 2011). Individual fish counts were done up to 20 individuals. The abundance of schooling species, for example, the bogue, *Boops boops*, was estimated using abundance classes: 21–30, 31–40, 41–50, 51–100, 101–200, and 201–400, based on a modification of the method presented by Harmelin‐Vivien et al. (1985). Seawater visibility ranged between 8 and 15 m in Gran Canaria Island; in southern Portugal, however, the visibility decreased from the “outer” (5–10 m) to the “inner” (3–5 m) side of the marinas. To avoid bias, fish counts were not performed if visibility was <3 m. To minimize overestimation of highly mobile species, while underestimating cryptic species (Lincoln‐Smith, 1988), each transect was performed twice. First, the diver swam at a constant speed, determined by the observer's natural swimming ability, annotating the abundance of noncryptic species. To minimize repeated counts of the same fish, the diver performed two instantaneous counts along the transect within a 5‐m length section, not counting fishes that overtook him (Labrosse, Kulbicki, & Ferraris, 2002). On the way back, the diver slowly swam, carefully searching for cryptic species in complex habitats (e.g., small caves, ledges, and overhangs). Fishes that were not visually identified to species level were recorded as genera; each fish was then treated as a distinct species in the statistical analyses.

#### 2.2.2 Fish traps

We used circular, wire‐framed, fish traps (15 mm of mesh size, 82.5 cm of inner diameter × 53 cm height), including two funnel entrances and a door at the top to add bait and extract catches (Appendix S1). At Gran Canaria Island, a total of 24 samples were taken for each marina, including 12 replicated traps per side and marina. However, in southern Portugal, the sampling effort was lower with six replicate traps per side. Each trap was baited with fresh Atlantic chub mackerel (*Scomber colias*) in Gran Canaria Island, and a mix of Atlantic chub mackerel, mussels (*Mytilus edulis*) and sardine oil (*Sardina pilchardus*) in southern Portugal. Adjacent traps were 10–15 m apart. Traps were dropped and subsequently retrieved after 2.5 hrs (Bacheler et al., 2013; Harvey et al., 2012). All collected fishes were identified to the lowest taxonomic level and then released.

#### 2.2.3 Baited cameras

Single underwater baited cameras (Appendix S2) were placed horizontally on the seabed, as similarly reported by Cappo, Harvey, Malcolm, and Speare (2003). The system consists of a main horizontal bar, which supports two vertical secondary bars, both made of stainless steel, one containing a PVC container (20 cm length × 10 cm height) with the bait, and the other with the camera (Gopro Hero 3+). A separation of 1 m between the bait and the camera was established to optimize the focus (field view), as fishes approach to the camera. At both ends of the main bar, we placed stabilizing arms, to ensure the device landed horizontally on the seafloor. To increase the dispersion of the odor plume, the bait container was elevated above the seafloor.

At each side of each marina, three replicated BCs were placed sequentially on the seafloor and recovered after 45 min, in the same sequential order. Filming times between 25 and 30 min have been reported as adequate for obtaining accurate relative abundances of fish species (Langlois et al., 2010; Stobart et al., 2007). Once recovered, the BC units were randomly relocated at different places within the same side, for a total of 60 deployments and 27 hrs of video recording for the whole study. Adjacent BCs were between 10 and 15 m apart. Each bait container was filled with the same bait as the traps. From each 45 min video recording, we extracted 1‐minute photo frames (at 10 MP) to assess the relative abundances of fish species; these were used to derive the maximum number of each species in the field of view in a single frame for each sample (MaxN), as a conservative measure of the relative abundances of species (Cappo et al., 2003; Willis & Babcock, 2000). To avoid bias, particularly in the case of southern Portugal, where visibility conditions were greatly reduced from the “outer” to the “inner” sides of marinas, fish counts were only performed for those individuals present in the field of view between the camera and the bait canister (1 m). The freeware ImageJ was used to count individuals of each species present in each photo frame, using the cell counter plugin. In some cases, due to the poor quality of the images, the brightness and contrast had to be increased/decreased to facilitate fish identification. Fishes that were not identified to the level of species were recorded as genera; each was then treated as a distinct taxon in the statistical analyses.

#### 2.2.4 Sampling method comparisons

Species abundance data were transformed to presence/absence to explore similarities among sampling methods in terms of community composition. Venn diagrams were generated to observe the overlap in the composition of fish faunas between the three sampling techniques. We initially tested for similarities between each pair of sampling techniques. We calculated Jaccard similarities between all pairs of samples provided by the different sampling techniques at each side of the marinas, which were then averaged to obtain a mean similarity between each pair of techniques at each region. Differences in similarities were tested through a one‐way ANOVA, including the factor “Method” (fixed factor with three levels: UVC‐BC, BC‐FT, and FT‐UVC). The assumption of homogeneity of variances was checked by means of the Cochran's test. When this was violated, data were ln (*x* + 1)‐transformed. If homogeneity of variance was still violated after transformations, the alpha value was set to .01, to decrease the probability of a type I error occurring (Underwood, 1997). Alternatively, we conducted a PCO to explore how much of the variation in community composition could be attributed to “Method” versus “Side” (these analyses can be found in https://www.researchgate.net/profile/Nestor_Bosch). Finally, we computed the mean overall relative abundance (i.e., data pooled across sides and marinas) of each fish species provided by each sampling technique at each ecoregion. We then investigated the relationships in mean overall species relative abundances between pairs of sampling methods (i.e., paired for all the species) using Pearson's correlation coefficients. ANOVAs, Pearson's correlations, and Venn diagrams were carried out in R v3.2.3; the package “VennDiagram” v.1.6.17 was used for the latter analysis.

#### 2.2.5 Biodiversity patterns

Three biodiversity indexes were calculated for each replicated sample provided by each method: (1) taxonomic diversity (TD; Shannon–Wiener diversity index, *H*′), (2) phylogenetic diversity (PD; taxonomic distinctness index, Δ*; Clarke & Warwick 2001), and (3) functional diversity (FD; Rao index of diversity, adapted for functional diversity; Botta‐Dukát, 2005). *H*′ is the most common measure of taxonomic diversity, accounting for both species richness and equitability: *H* ^′^ = −∑*i* ρ*i* log(ρ*i*); where ρ*i* is the proportion of the total count for the *i*th species. Δ* is based not only on species abundances, but also in the taxonomic distances (ω_*ij*_) between every pair of species, following the standard Linnean classification tree. This index is derived by dividing the average taxonomic diversity (Δ) by the Simpson index (Δ°) (Clarke & Warwick 2001) and takes the form of: $\Delta^{*}=\frac{\left[\sum \sum_{i<j}\omega_{ij}x_{i}x_{j}\right]}{\sum \sum_{i<j}x_{i}x_{j}}$; where the double summation is over all pairs of species *i* and *j*, and *x*_*i*_ and *x*_*j*_ are the total numbers of individuals of the *i*th and *j*th species in the sample. Finally, the Rao index uses species traits to calculate dissimilarities among species: $\text{FD}=\sum_{i=1}^{S}\sum_{j=1}^{S}d_{ij}p_{i}p_{j}$; where *S* is the number of species, *p*_*i*_ and *p*_*j*_ are the proportion of *i*th and *j*th species, and *d*_*ij*_ is the dissimilarity between species *i* and *j*, which varies between 0 (two species have exactly the same traits) and 1 (two species have completely different traits).

The Rao index was computed for each trait and then averaged for each sample across all traits together. Seven functional traits were considered: trophic niche and breadth, maximum body length and shape, behavior, habitat associations, and life history characteristics (Table 1). Maximum length, trophic breadth, and trophic level were included as continuous traits and scaled between 0 (minimum) and 1 (maximum), while the rest of the traits were categorical. Trophic groups were established according to Tuya, Boyra, Sanchez‐Jerez, Barbera, and Haroun (2004). Most values and attributes were compiled from Fishbase (www.fishbase.org; Froese & Pauly, 2002), but also from existing literature. When information on specific species was not available, we used values from sibling species, often within the same genus and geographic area. All three indexes were calculated on square‐root‐transformed data. *H*′ and Δ* were calculated using the PRIMER 6 software (Clarke & Warwick, 2001), while the Rao index of functional diversity was calculated using the Macro excel file (“FunctDiv.exl”) (Lepš, De Bello, Lavorel, & Berman, 2006).

**Table 1:** Functional traits for each fish species, adapted from Micheli and Halpern (2005) and Stuart‐Smith et al. (2013)

Functional trait	Category	Type	Units
Maximum length	Body size	Numerical	Total length (cm)
Trophic breadth	Trophic niche	Numerical	Number of prey phyla consumed (from diet studies). Range from 1 to 8
Trophic group	Trophic niche	Categorical	Planktivorous, Omnivorous, Herbivorous, Micro‐invertebrate feeders, Macroinvertebrate feeders, Macroinvertebrate feeders and piscivorous
Water column position	Behavior	Categorical	Benthic, bentho‐pelagic, and pelagic
Preferred substrate	Habitat use	Categorical	Hard bottoms and soft bottom
Trophic level	Trophic niche	Numerical	Index, range from 1 to 5
Body shape	Body shape	Categorical	Fusiform, compressed, depressed, globiform, and elongated

A two‐way crossed ANOVA tested, separately for each biodiversity index and sampling method, for differences between “Side” (fixed factor) and “Marina” (random factor orthogonal to “Side”), following the criteria previously specified. When significant “Marina × Side” interactions were found, a pairwise test was used to resolve differences between the “inner,” “middle” (exclusively for southern Portugal), and “outer” sides of each marina.

#### 2.2.6 Sampling effort comparisons

At each region, we estimated the costs (per sample) by UVC, BC, and FT. The costs were expressed in staff time, as this simplifies the comparisons between regions and can be easily translated into future monitoring programs. General costs including program management, equipment, mobilization, insurance, and consumables were not included in the analysis, as these are specific to a research program. The time to set up and break down the equipment ranged between 15 and 20 min, and two field scientists were required to carry out each survey. As these were comparable between methods, we excluded this information from the analysis.

The adequacy of the sampling effort by the three sampling techniques to assess fish assemblages at each side (i.e., data pooled for marinas) within each region was firstly assessed through species accumulation curves via EstimateS v.9.00 (Colwell, 2013). Sample‐based rarefactions through 100 randomizations of the samples were selected (Colwell, Mao, & Chang, 2004).

Secondly, we computed the maximum number of species observable by each method (*S*_max_) at each side within each eco‐region, as well as the number of samples (*m*) required to reach a target proportion (*g*) of the asymptotic richness, using the Excel spreadsheet tool developed by Chao, Colwell, Lin, and Gotelli (2009). This procedure uses the abundances of the rarest species, that is, species observed in only one (“uniques”) or two (“duplicates”) samples, to estimate the frequencies of undetected species, which is then used to provide an estimation of the asymptotic richness of an assemblage, computed using the Chao 2 nonparametric estimator. This has proved to be a robust estimator of the minimum species richness of an assemblage, as well as being less biased for small sample sizes (Colwell & Coddington, 1994; Shen, Chao, & Lin, 2003). The number of additional samples required to achieve a certain proportion of the Chao 2 asymptotic richness (*mg*) is then computed using the formula: $mg=\frac{\text{log}\left[1-\frac{t}{\left(t-1\right)}\frac{2Q_{2}}{Q_{1}^{2}}{(}_{g}S_{\mathrm{est}}-S_{\mathrm{obs}})\right]}{\text{log}\left[1-\frac{2Q_{2}}{\left(t-1\right)Q_{1}+2Q_{2}}\right]}$, where *S*_obs_ is the observed species richness, _g_ *S*_est_ is the predicted species richness for a target fraction of the asymptotic richness based on Chao 2 and must be >*S*_obs_, *t* is the number of samples collected, *Q*_1_ is the number of “uniques,” and *Q*_2_ is the number of “duplicates.” Finally, the number of samples required to achieve a proportion of .90 and .95 of the maximum species richness available by that method, at each side within each region, was multiplied by the mean time per replicate sample for each method to produce a standardized metric, which allowed us to account for between‐methods differences in sampling effort.

Thirdly, we explored the power of each method to detect a change (“effect size”) of 25% and 50% with increasing sample size, using the mean and variances estimates of the three biodiversity indexes for each region (pooled for sides and marinas). A one‐way ANOVA with two levels, “inner” versus “outer,” was used to calculate noncentral *F* probabilities for each comparison using the program G*Power (Faul, Erdfelder, Lang, & Buchner, 2007), as done by Langlois et al. (2010). We used the information on the cost per sample to calculate the effort that would be required to achieve a power of 0.8 for each variable.

## 3 Results

### 3.1 Sampling methods comparisons

**Figure 2:**
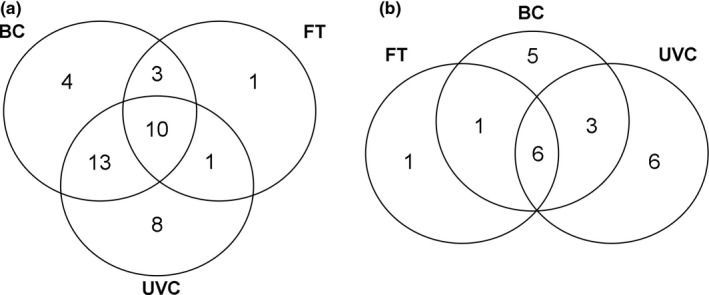
Venn diagrams showing the overlap in fish species composition recorded by the different sampling methods: Underwater visual census (UVC), baited camera (BC) and fish trap (FT) at Gran Canaria Island (a) and southern Portugal (b)

Overall, we observed 40 fish species at Gran Canaria Island, 23 of commercial relevance (González et al., 2012); in southern Portugal, we registered 22 species, 10 commercially relevant (Borges et al., 2001). At both regions, UVCs and BC recorded a comparable number of species (32 vs. 30 at Gran Canaria Island; 15 at southern Portugal, respectively) and families (18 vs. 15 at Gran Canaria Island; 9 vs. 6 at southern Portugal, respectively) (Appendices S3 and S4). FT collected the lowest number of species (15 at Gran Canaria Island and 9 at southern Portugal) and families (8 and 6, respectively) (Appendices S3 and S4). At both regions, UVC‐BC shared the greatest number of species, followed by BC‐FT, and finally UVC‐FT, which shared the lowest number (Figure 2a,b).

Species that were recorded by BC and/or FT, but not through UVC, included piscivorous species, such as the brown moray (*Gymnothorax unicolor*), the moray (*Muraena augusti*), the blacktail comber (*Serranus atricauda*), and the dusky grouper (*Ephinephelus marginatus*), at Gran Canaria Island (Appendix S3); the European conger (*Conger conger*), the European sea bass (*Dicentrarchus labrax*), and the Mediterranean moray (*Muraena helena*), at southern Portugal (Appendix S4). Conversely, UVC recorded the largest number of cryptic species, 6 at Gran Canaria Island and 8 at southern Portugal, including the redlip blenny (*Ophioblennius atlanticus*), the ringneck blenny (*Parablennius pilicornis*), the blenny (*Parablennius* sp.1), the black goby (*Gobius niger*), the rock goby (*Gobius paganellus*), and the goby (*Gobius xantocephalus*), at Gran Canaria Island (Appendix S3); the red‐mouthed goby (*Gobius cruentatus*), *G. niger*,* G. paganellus*,* Gobius* sp.1, *G. xantocephalus*, the tompot blenny (*Parablennius gattorugine*), *P. pilicornis*, and the black‐faced blenny (*Tripterygion delaisi*), at southern Portugal (Appendix S4).

**Figure 3:**
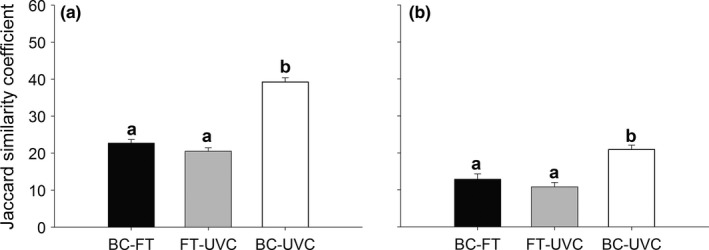
Mean (+*SE*) Jaccard similarities in the composition of fish assemblages between pairs of sampling methods for Gran Canaria Island (a) and southern Portugal (b). Different letters above bars denote statistically significant differences

At both regions, similarities in fish assemblage composition varied between sampling methods (Figure 3a,b; “Method”: *F*_2,717_ **= 42.76, *p *<* *.001, at Gran Canaria Island; “Method”: *F*_2,645_ = 17.66, *p *<* *.001, at southern Portugal). Pairwise tests showed a larger similarity in the composition of fish faunas between UVC and BC relative to BC‐FT (*t*_430_ = 7.91, *p *<* *.001, at Gran Canaria Island; *t*_430_ = 4.47, *p *<* *.001, at southern Portugal) and FT‐UVC (*t*_430_ = 8.84, *p *<* *.001, at Gran Canaria Island; *t*_430_ = 5.62, *p *<* *.001, at southern Portugal). In terms of fish abundances recorded by each sampling method, significant Pearson's correlations were found between all sampling methods at Gran Canaria Island. The highest correlation was found between BC and UVC (*r *=* *.90, *p *<* *.001), followed by BC and FT (*r *=* *.85, *p *<* *.001), and FT and UVC (*r *=* *.61, *p *=* *.04). However, at southern Portugal, no significant correlations were found between the sampling methods (BC‐UVC, *p *=* *.1; BC‐FT, *p *=* *.79; FT‐UVC, *p *=* *.90).

### 3.2 Biodiversity patterns

**Figure 4:**
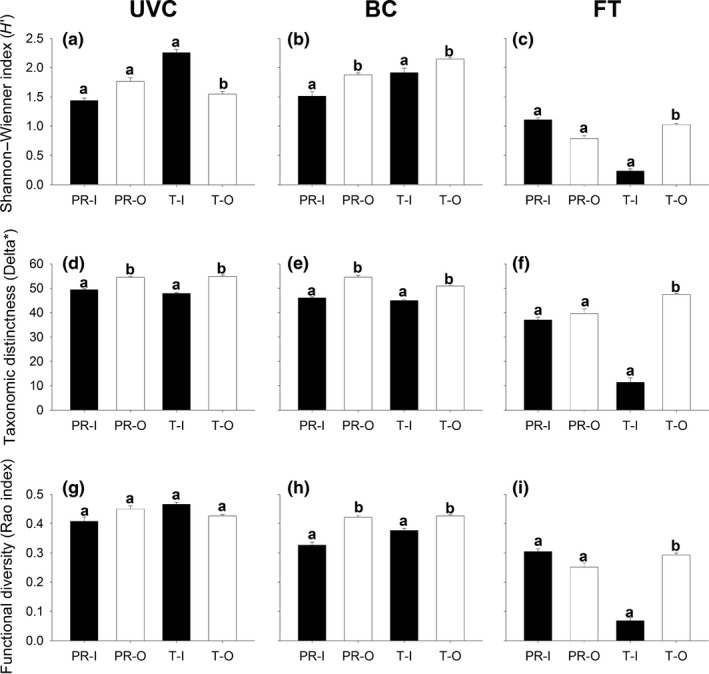
Mean (+*SE*) diversity, for each sampling method (UVC, left; BC, middle; FT, right), according to the Shannon–Wienner diversity index (*H*′) (a–c), the taxonomic distinctness (∆*) (d–f) and the Rao index of functional diversity (g–i) at each side (I = “inner,” black bars; O = “outer,” white bars) of each marina (PR = Puerto Rico; T = Taliarte) from Gran Canaria Island. Different letters above bars denote statistically significant differences. UVC, underwater visual census; BC, baited camera; FT, fish trap

**Figure 5:**
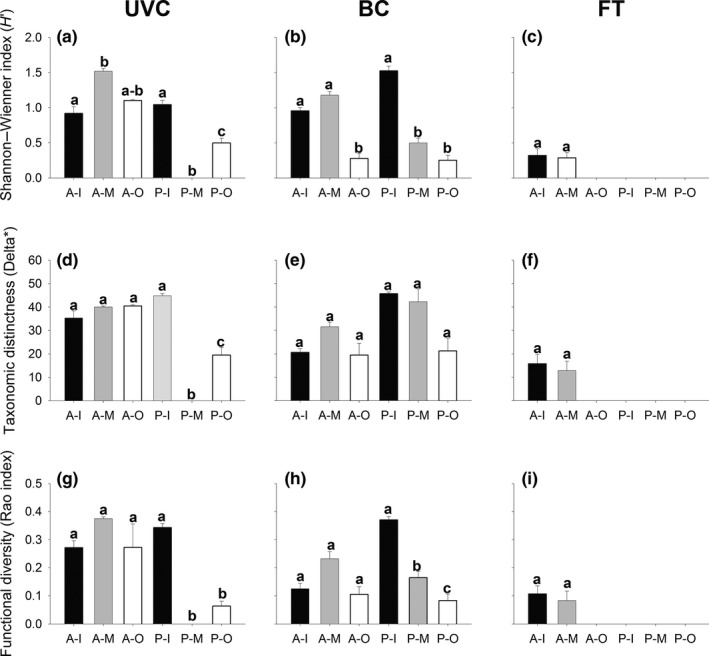
Mean (+*SE*) diversity, for each sampling method (UVC, left; BC, middle; and FT, right), according to the Shannon–Wienner diversity index (*H*′) (a–c), the taxonomic distinctness index (∆*) (d–f) and the Rao index of functional diversity (g–i) at each side (I = “inner,” black bars; M = “middle,” gray bars; O = “outer,” white bars) of each marina (A = Albufeira; P = Portimão) from southern Portugal. Different letters above bars denote statistically significant differences. UVC, underwater visual census; BC, baited camera; FT, fish trap

At both regions, patterns of biodiversity of reef fishes between sides of the marinas generally varied between sampling methods and metrics (Figures 4 and 5). Overall, at Gran Canaria Island, consistently significant differences in TD between the “inner” and “outer” sides were found in BC data set (Figure 4b, “Side,” *p *<* *.05, Table 2); however, for UVC and FT, these differences varied from marina to marina (Figure 4a,c, “Marina × Side,” *p *<* *.05, Table 2). Similarly, in southern Portugal, differences in TD between sides varied from marina to marina (Figure 5a–c, “Marina × Side,” *p *<* *.05, Table 3) for UVC and BC data, while no significant difference in TD was found for FT data (Figure 5c). In addition, we found inverse patterns depending on the sampling method used. For example, at Taliarte, BC found greater TD at the “outer” side, while TD was greater at the “inner” side for UVC and FT (Figure 4a–c, p* *<* *.05, Table 2). Interestingly, overall, PD was consistently higher in the “outer” sides at Gran Canaria Island for all sampling methods (Figure 4d,f,g, “Side,” *p *<* *.05, Table 2), although this pattern was not significant in the case of Puerto Rico for FT (Figure 4f, “Marina × Side,” *p *<* *.05, Table 2). Conversely, at southern Portugal, PD did not show any consistent pattern, and no significant differences between sides were found for the three sampling methods (Figure 5d). At both regions, the Rao index (FD) showed a similar pattern to the Shannon–Wiener index (TD), suggesting a close association between these metrics, although in some cases, the Rao index failed to detect significant changes found by the Shannon–Wienner index and vice versa (Figures 4 and 5g,h,d, Tables 2 and 3). We must be cautious when interpreting the significance of these results, as the power of the test was low in some cases (Appendix S6), although this does not affect the overall differences in diversity patterns depending on the sampling method used.

**Table 2:** Two‐way crossed ANOVA testing for differences in fish diversity between “Side” (Si, fixed factor) and “Marina” (Ma, random factor) according to each sampling method at Gran Canaria Island

	UVC										BC									FT											
	*H*′				Δ*			Rao			*H*′			Δ*			Rao			*H*′				Δ*				Rao			
Cochran test	No transformation *p* = .81 (n.s.)				No transformation *p* = .12 (n.s.)			No transformation *p* = .10 (n.s.)			No transformation *p* = .26 (n.s.)			No transformation *p* = .09 (n.s.)			No transformation *p* = .24 (n.s.)			No transformation *p* = .17 (n.s.)				No transformation*p* = .052 (n.s.)				No transformation *p* = .056 (n.s.)			
Source	*df*	MS	*F*	*p*	MS	*F*	*p*	MS	*F*	*p*	MS	*F*	*p*	MS	*F*	*p*	MS	*F*	*p*	*df*	MS	*F*	*p*	MS	*F*	*p*		MS	*F*	*p*	
Ma	1	0.54	5.8	**.025**	2.1	0.2	.6	1e^−3^	0.27	.6	0.6	5.4	**.03**	0.16	4.3	**.04**	4e^−3^	2.2	.14	1	0.7	5.7	.06	21	3.6	.06		0.2	6.07	**.02**	
Si	1	0.22	2.3	.13	215	26	**<.001**	6e^−4^	0.01	.8	0.5	4.34	**.05**	1.59	42	**<.001**	0.03	15	**7e** ^−**4**^	1	0.8	5.9	**.01**	70	11	**.001**		0.2	6.65	**.01**	
Ma × Si	1	1.60	17	**4e** ^−**4**^	5.1	0.6	.4	5e^−3^	1.56	.2	0.02	0.20	.6	0.03	1.0	.27	3e^3^	1.5	.23	1	3.0	22	**<.001**	94	15	**<.001**		0.7	17.9	**<.001**	
Residual	20	0.09			8.1			3e^−3^			0.12			0.03			0.001			44	0.1			5.9				4e^−3^			
Pairwise tests	PR		TA																	PR		TA		PR		TA		PR		TA	
	*t*	*p*	*t*	*p*																*t*	*p*	*t*	*p*	*t*	*p*	*t*	*p*	*t*	*p*	*t*	*p*
I versus O	1.8	.08	4	**<.001**																1	.1	5	**<.001**	0.3	.7	5	**<.001**	1.1	.24	4.8	**<.001**

Pairwise tests are also included. PR = Puerto Rico; TA = Taliarte. I = “inner”; O =  “outer.” Significant values are highlighted in bold. Significance level (α) = .05; when homogeneity of variance was still violated despite transformation α = .01.

**Table 3:** Two‐way crossed ANOVA testing for differences in fish diversity between “Side” (Si, fixed factor) and “Marina” (Ma, random factor) according to each sampling method at southern Portugal

	UVC												BC												FT											
	*H*′				Δ*				Rao				*H*′				Δ*				Rao				*H*′				Δ*				Rao			
Cochran test	Ln (*x* + 1) ***p*** ** < .05**				Ln (*x* + 1) ***p*** ** < .05**				Ln (*x* + 1) ***p*** ** < .05**				No transformation *p* = 1.39 (n.s.)				No transformation *p* = .40 (n.s.)				No transformation *p* = 1.02 (n.s.)				Ln (*x* + 1) ***p*** ** < .05**				Ln (*x* + 1) ***p*** ** < .05**				Ln (*x* + 1) ***p*** ** < .05**			
Source	*df*	MS	*F*	*p*	MS	*F*	*p*		MS	*F*	*p*		MS	*F*	*p*		MS	*F*	*p*		MS	*F*	*p*		MS	*F*	*p*		MS	*F*	*p*		MS	*F*	*p*	
Ma	1	3.99	34.44	**<.001**	3.90	22.63	**<.001**		0.26	32.5	**<.001**		0.01	0.12	.7		1,408	2.51	.12		0.02	1.3	.2		0.36	4.14	.04		815	4.14	.05		0.03	3.1	**.08**	
Si	2	0.16	1.434	.09	1.55	9	**<.001**		0.06	8.44	**<.001**		2.89	19.6	**<.001**		907	1.61	.21		0.07	4.2	**.02**		0.09	1.04	.3		210	1.06	.3		0.009	0.81	.4	
Ma × Si	2	2.03	17.51	**<.001**	2.73	15.87	**<.001**		0.15	18.9	**<.001**		1.17	8	**<.001**		414	0.73	.48		0.08	4.8	**.01**		0.09	1.04	.3		210	1.06	.3		0.009	0.81	.4	
Residual	30	0.11			0.17				0.008				0.14				560								0.08				196				0.01			
Pairwise tests	A		PO		A		PO		A		PO		A		PO						A		PO													
	*T*	*p*	*T*	*p*	*T*	*p*	*T*	*p*	*T*	*p*	*T*	*p*	*T*	*p*	*T*	*p*					*T*	*p*	*T*	*p*												
M versus I	3.05	**.005**	5.29	**<.001**	1.04	.3	6.92	**<.001**	1.99	.06	6.61	**<.001**	1.01	.3	4.64	**<.001**					1.39	.17	2.69	**.01**												
M versus O	2.11	.06	2.77	**.009**	0.01	.9	3.99	**<.001**	1.97	.6	1.22	.2	4.07	**<.001**	1.11	.27					1.66	.10	1.06	.29												
O versus I	0.93	0.3	2.52	**0.01**	1.06	.2	2.92	**.006**	0.02	.9	5.38	**<.001**	3.06	**.005**	5.75	**<.001**					0.26	.79	3.75	**<.001**												

Pairwise tests are also included. A = Albufeira; PO = Portimão. I = “inner”; M = “middle”; O = “outer.” Significant values are highlighted in bold. Significance level (α) = .05; when homogeneity of variance was still violated despite transformation α = .01.

### 3.3 Sampling effort comparisons

Each UVC field survey (per side of each marina) took an average of 45 min per side, including 1 min to reach the bottom, 5 min to lay the transects on the seafloor, a 1‐min pause before the start of the censuses to reduce bottom disturbance, 5 min to retrieve the transects, and a 3 min safety stop before ascending to the surface. The mean field cost per UVC sample was 5–6 min, varying as a function of the number and abundances of species present (Table 4). BC surveys consisted of three BC units concurrently deployed for 47 min, including 1 min to reach the bottom and account for bottom disturbance, 1 min for retrieval for each unit, and 45‐min video of recording (Table 4). Within each side at each marina, this procedure was repeated giving a total field time (per side) of 102 min. Finally, FT required the greater amount of field time, consisting of a soak time of 150 and 2 min for launch and retrieval for each of 6 traps (Table 4). At Gran Canaria Island, this procedure was repeated within each side giving a total field time per side of 324 min. For each FT sample, we estimated that an average of 5 min was required to identify and count fish species; therefore, the final estimated total field times per side were 384 min at Gran Canaria Island and 192 min at southern Portugal.

**Table 4:** Summary of the estimated field and laboratory costs per sample using underwater visual census (UVC), baited cameras (BCs), and fish traps (FTs), for Gran Canaria Island (GC) and southern Portugal (SP)

	Field time (min)	Laboratory time (min)		Total (min)	
	GC‐SP	GC	SP	GC	SP
UVC	5	3	3	11	11
BC	45	90	60	105	75
FT	150	3	3	32	33

Field and laboratory costs are expressed as staff time (min).

Laboratory time differed greatly between sampling techniques. We estimated that it takes ~20 min (per side) to enter the data collected in an UVC and FT survey in an excel sheet (Table 4), while the average time taken to analyze 45 image footages extracted from each BC sample, and enter the data in an appropriate excel sheet, was estimated to be 90 min at Gran Canaria Island and 60 min at southern Portugal. Therefore, the estimated mean total laboratory time (per side) for BC was 540 min at Gran Canaria Island and 360 min at southern Portugal. Differences between regions resulted from differences in number and abundance of species, and the ease by which they could be identified (Cappo et al., 2003). Using these times, we estimated that the total mean time per replicate was 11 min for UVC, 105 and 75 min for BC at Gran Canaria Island and southern Portugal, respectively, and 33 min for FT (Table 4). Note that these estimates are based on the specific settings of our research program, and thus, any increase in the number of BC and FT units that could be deployed concurrently will reduce the mean time per replicate.

**Figure 6:**
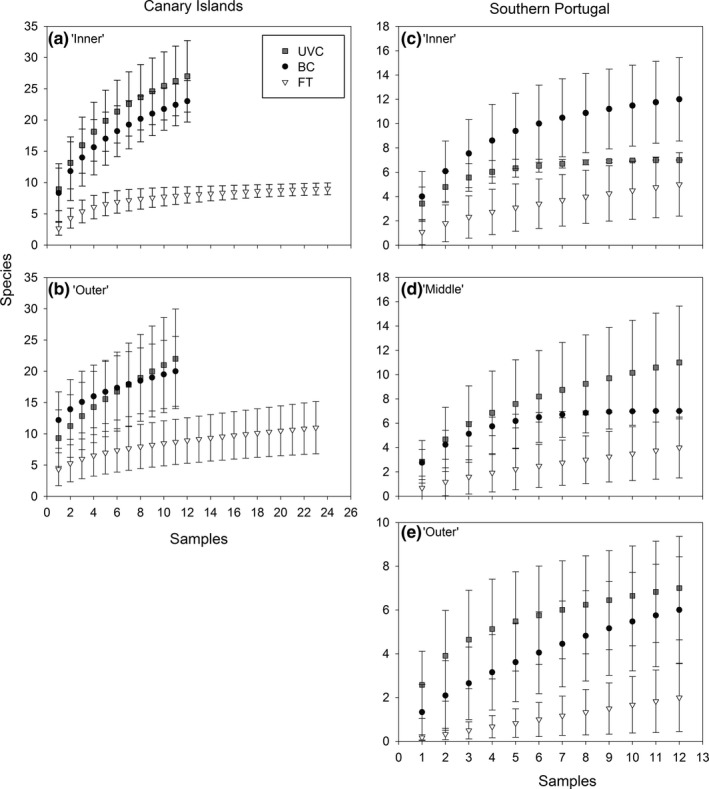
Species accumulation curves (sample‐based rarefaction), for each method at each side, at Gran Canaria Island (left column) and southern Portugal (right column). Values are expected species richness ±95% confidence intervals

Species accumulation curves showed that the effort to account for the complete fish assemblage was generally insufficient, as the curves rarely reached an asymptote (Figure 6). However, the methods overall sampled a relatively high proportion of *S*_max_, estimated by Chao 2 (Appendix S5). At all sides, but the “inner” at southern Portugal, the estimated number of species (*S*_obs_) was higher using UVC than BC, although these differences were not statistically significant, except for the case of the “inner” sides at southern Portugal (Figure 5c). FT recorded a significantly lower species richness at all sides, although these differences were less pronounced at southern Portugal (Figure 5a–c). The same pattern was observed for the estimated Chao 2 asymptotic richness (Table 5). However, as sampling units are inherently different among sampling methods, we must be cautious when drawing conclusions from these comparisons. At Gran Canaria Island, we found that more UVC than BC samples would be required to observe the same proportion of *S*_max_ available, while FT required the largest number of samples independently of the side (Table 5). At southern Portugal, the number of samples required varied from side to side (Table 5). At the “inner” and “outer” sides, more BC than UVC samples were required, while more UVC than BC samples were required at the “middle” sides. Again, FT required the largest number of samples. However, after standardizing by the sampling effort, we found that UVC consistently (i.e., across sides and regions) needed less effort to achieve a standardized proportion of the Chao 2 asymptotic richness, while BC and FT required a considerably greater effort (Table 5).

**Table 5:** Predicted maximum species richness (*S*_max_) determined from species accumulation curves, total number of samples (*m*), and effort (*h*) required to reach a proportion (*g*) of .90 and .95 of the Chao 2 asymptotic species richness for each method at each side within each region

	*S*_max_ ± *SE*			Number of samples required (*m*)						Effort (*h*) required					
	UVC	BC	FT	*g* = .90			*g* = .95			*g* = .90			*g* = .95		
Gran Canaria Island				UVC	BC	FT	UVC	BC	FT	UVC	BC	FT	UVC	BC	FT
Inner	30 ± 2.39	25 ± 2.33	9 ± 0.27	7	**6**	12	12	**11**	12	**1.3**	10.5	6.4	**2.2**	19.3	6.4
Outer	27 ± 3.51	22 ± 1.22	19 ± 2.28	13	**6**	77	21	**11**	109	**2.4**	10.5	41.1	**3.9**	19.3	58.1
Southern Portugal															
Inner	7 ± 0.13	13 ± 0.76	6 ± 0.53	**6**	**6**	11	**6**	8	18	**1.1**	7.5	6.1	**1.1**	10.0	9.9
Middle	13 ± 1.21	7 ± 0.23	5 ± 0.45	11	**6**	16	18	**6**	24	**2.0**	7.5	8.8	**3.3**	7.5	13.2
Outer	8 ± 0.35	7 ± 0.66	3 ± 0.21	**6**	9	17	**9**	15	21	**1.1**	11.3	9.4	**1.7**	18.8	11.6

The most cost‐efficient sampling method is highlighted in bold.

**Figure 7:**
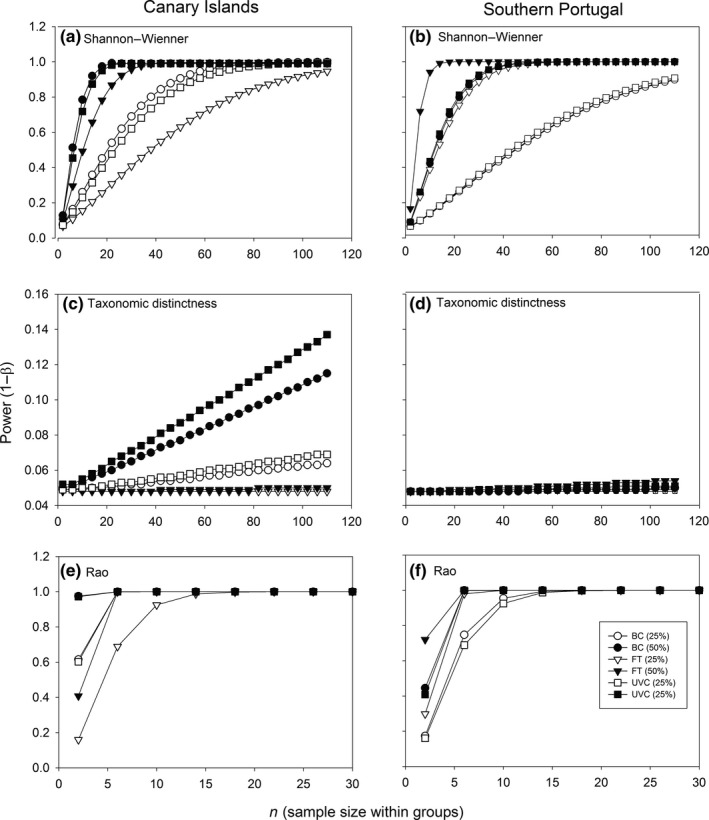
Varying statistical power to a detect a change (“effect size”) of 25% and 50% for the Shannon–Wienner index (a,b), the taxonomic distinctness index (c,d), and the Rao index (e,f) at Gran Canaria Island (left column) and southern Portugal (right column), according to each sampling technique

Overall, UVC and BC shared a similar power to detect significant changes at comparable levels of replication for the biodiversity indexes, except for the taxonomic distinctness index at Gran Canaria Island, where the power was greater for UVC (Figure 7). However, in this case the power was very low, even at high levels of replication for both regions. UVC and BC had consistently (i.e., across biodiversity indexes) more statistical power at Gran Canaria Island, while at southern Portugal we observed an inverse pattern (Figure 7), probably resulting from the high number of 0s found in FT samples at southern Portugal. The Rao index was the most robust metric (i.e., with the smallest variation among replicated samples), as the power was high even at low levels of sampling replication, followed by the Shannon–Wienner index, and finally the taxonomic distinctness index, which was the most sensitive metric. After standardization by sampling effort, UVC was found to be the most cost‐efficient technique, as it consistently (i.e., across indexes and regions) required the least amount of effort to achieve a power of 0.8 (Table 6). The only exception was found in the case of the Shannon–Wienner index at southern Portugal, where FT required less effort (Table 6). The cost‐efficiency of the different methods for the taxonomic distinctness index was not assessed, as the number of replicates and effort required to detect a change of 25% and 50% were logistically not feasible (Table 6).

**Table 6:** Cost‐efficiency of different sampling techniques at each region to detect a change of 25% and 50% for the Shannon–Wienner, taxonomic distinctness index and Rao index

Index	Region	Method	Replicates to detect a change of		Effort (*h*) to detect a change of	
			25%	50%	25%	50%
Shannon–Wienner	Gran Canaria Island	**UVC**	44	12	**8.1**	**2.2**
		BC	**37**	**10**	64.8	17.5
		FT	70	19	37.3	10.1
	Southern Portugal	UVC	83	22	15.2	4.0
		BC	86	20	107.5	25.0
		**FT**	**24**	**7**	**13.2**	**3.9**
Taxonomic distinctness	Gran Canaria Island	UVC	>120	>120	>210	>210
		BC	>120	>120	>210	>210
		FT	>120	>120	>210	>210
	Southern Portugal	UVC	>120	>120	>210	>210
		BC	>120	>120	>210	>210
		FT	>120	>120	>210	>210
Rao	Gran Canaria Island	**UVC**	**4**	**2**	**0.7**	**0.4**
		BC	**4**	**2**	7.0	3.5
		FT	8	4	4.3	2.1
	Southern Portugal	**UVC**	8	5	**1.5**	**0.9**
		BC	7	5	8.8	6.3
		FT	**5**	**3**	2.8	1.7

Values are derived from the power analysis at the point where the curve reaches a 0.80 power. The most cost‐efficient technique at each region is highlighted in bold. For logistical reasons, the number of replicate samples and effort was limited to 120 and 210 hr, respectively.

## 4 Discussion

The ability of different survey methods to complement each other and provide accurate, rapid, and cost‐effective data is essential (Baker et al., 2016; Langlois et al., 2010). In our work, we found that UVCs and BCs recorded a comparable number of species, as observed in the Venn diagrams, species accumulation curves, and measures of asymptotic richness, while FT registered a considerably lower number. Some studies have reported that UVC accounted for a wider range of species than BC, as a diverse set of habitats types may be rapidly sampled, with both pelagic and benthic species recorded (Colton & Swearer, 2010; Stobart et al., 2007). However, other studies have observed a greater number of species from BC than from diver‐based methods (Langlois et al., 2010; Watson et al., 2005; Willis & Babcock, 2000). These contrasting results are not surprising, as studies have been carried out in different regions, under different environmental conditions, and sampling designs. The high similarity in the composition of fish faunas between UVC and BC reported here suggest that these techniques provide complementary information. Furthermore, the high correlation in mean overall relative abundances of fish species between UVC and BC, at Gran Canaria Island, suggests that these methods not only sampled a similar part of the fish assemblage, but also in similar relative abundances. Conversely, no correlation in mean overall relative fish abundances between UVC and BC was found in southern Portugal, probably as a result of adverse environmental conditions during winter, namely high turbidity and high hydrodynamics, which compromise the performance of visual techniques (MacNeil et al., 2008; Murphy & Jenkins, 2010), resulting in low abundances of most species. The high selectivity of FT is well known, which results in many zero records, especially in the case of nontarget species not attracted to the bait (Bacheler et al., 2013; Harvey et al., 2012). This explains the low similarity in species composition between this technique and UVC and BC.

The extent to which different survey methods affect biodiversity estimates has received little attention and represents an important topic in diversity research, particularly in the marine realm (Robinson et al., 2014). Overall, we found distinct patterns of TD, PD, and FD between sides of the marinas, at both regions, depending on the sampling method to survey fish faunas. Patterns of fish biodiversity were more similar between UVC and BC than between BC and FT and UVC and FT; this is consistent with similarities in fish composition between techniques, as previously discussed. UVC and BC yielded greater values of TD, PD, and FD than FT at both regions. Differences in fish biodiversity patterns between sampling methods might be attributed to biases associated with the behavior and the ecological niche of species. In our work, we found certain piscivorous species that were detected by BC and FT, but not by UVC; in contrast, UVC recorded a larger number of cryptic species. Previous studies have found UVC to be more advantageous for recording cryptic species (Colton & Swearer, 2010; Stobart et al., 2007; Watson et al., 2005), while BCs have proved to be an effective method for sampling large mobile predatory species that usually avoid divers (Cappo et al., 2003; Langlois et al., 2010; Willis & Babcock, 2000). In fact, Lowry et al. (2012) demonstrated that species traits (i.e., behavior and life history) were the main drivers of variability in the frequency of detection of species between UVC and BC. We must be cautious when interpreting these results, as the statistical power was low in some cases (Appendix S6), although this does not affect the overall pattern of differences between methods.

Moreover, we found varying patterns of fish biodiversity between sides of the marinas depending on the level at which diversity was measured, that is, at the taxonomic, phylogenetic, or functional level. This is expected, as each index measures a distinct property of the fish assemblage; this has been described at a range of spatial scales. At global scales, Stuart‐Smith et al. (2013) found markedly different patterns of reef fish diversity when comparing functional (trait based) relative to taxonomic approaches. At local scales, Villéger et al. (2010) found contrasting changes in taxonomic and functional diversity of tropical fish communities following environmental degradation. These distinct patterns suggest that relationships between these measures of diversity can be of various types (Micheli & Halpern, 2005). In our study, we found that the patterns of fish diversity were similar for taxonomic and functional diversity, while phylogenetic diversity showed contrasting patterns, especially at Gran Canaria Island. Our results suggest a close association between TD and FD, thus reinforcing the idea that TD is a good surrogate of FD and vice versa (Leung, 2015; Wong & Dowd, 2015).

One fundamental aspect when comparing methods is the effect of sampling area and effort in community metrics, such as species richness (Gray, Ugland, & Lambshead, 2004). In our study case, although the sampling techniques were applied over a similar area, the actual area surveyed by each technique was different, as sampling units are different. This is particularly problematic in the case of species accumulation curves, and subsequent measures of asymptotic richness, as these might have underestimated differences between methods by treating samples of each method as equal. UVC allows an adequate estimation of the sampling area (Kulbicki et al., 2010), while the actual area surveyed by a single BC and FT is unknown. The development of stereo‐video techniques may overcome some the problems associated with standardization of the area sampled, as it allows establishing boundaries in the field of view (Colton & Swearer, 2010; Langlois et al., 2010). However, with BC and FT, the actual area surveyed is also a function of the dispersal range of the bait plume, as well as the sensory capacity, swimming speed, and behavior of species (Harvey, Cappo, Butler, Hall, & Kendrick, 2007). While some attempts to estimate the dispersal range of the odor plume have been made in deep waters (Heagney, Lynch, Babcock, & Suthers, 2007; Sainte‐Marie & Hargrave, 1987), and more recently in estuarine systems (Taylor, Baker, & Suthers, 2013), accounting for these distances in shallow water marine environments, subjected to complex hydrodynamic regimes, is problematic. In addition, in the case of FT, additional factors such as the species‐specific catchability and catch saturation effects also influence the number and abundance of species collected (Bacheler et al., 2013; Harvey et al., 2012). Therefore, differences between techniques may be attributed to differences in the area sampled. As shown in the species accumulation curves, however, any increase is likely to be nonlinear and unlikely to result in variation in the overall patterns (Langlois et al., 2010). This is one of the fundamental aspects to be evaluated when assessing the use of each technique.

Species accumulation curves and asymptotic richness estimators provide a useful tool to assess the degree to which different assemblages are adequately sampled, and so to provide guidelines for future allocation of resources to optimize monitoring programs (Chao et al., 2009; Gotelli & Colwell, 2001). Although the replication level by the three sampling techniques was similar, species accumulation curves differed, and rarely reached an asymptote. The achieved proportion of the asymptotic richness was overall higher, and close to the asymptote, at the “inner” sides, suggesting that the sampling effort here was sufficient (Appendix S5). At the “outer” sides, the proportion was lower, probably because of the presence of species from surrounding habitats with large moving capabilities. Our results initially suggested that, in general, more UVC than BC samples would be required to capture the same proportion of the asymptotic richness by each method, while FT would require the largest number of samples. After standardizing by sampling effort (i.e., taking into account the mean time per sample), UVC needed consistently less effort, and therefore can be perceived as a more efficient sampling technique to characterize fish species richness. Colton and Swearer (2010) found a similar trend, with UVCs being more efficient for species richness estimates than BC, after standardizing by sampling effort. However, we must note that estimators of asymptotic richness only provide lower bounds estimates of the “true” richness of an assemblage (Colwell et al., 2004). Furthermore, they are biased for small sample size, that is, tend to increase with reference sample size and often have large variance and confidence intervals (Gotelli & Colwell, 2011). Similarly, the number of additional samples required to reach a certain proportion of the asymptotic richness also increases with subsample size (Chao et al., 2009). Thus, our estimates may have underestimated the number of samples required for large proportions of the asymptotic richness and should only be considered conservative estimates.

Another way to evaluate the efficiency of sampling techniques, and so the allocation of time and resources to a sampling scheme, might be accounted by the statistical power (Millard & Lettenmaier, 1986). UVCs and BCs consistently had comparable statistical power, which were greater than FTs at Gran Canaria Island; at southern Portugal, we observed an inverse pattern, probably resulting from the high number of 0s in FT samples. In fact, the low mean values of diversity of this technique (i.e., low accuracy) suggest that it may be ineffective for most ecological studies looking at community wide patterns. At both regions, UVCs were the most cost‐efficient technique, as it required the least amount of effort to achieve a reasonable power. Contrary to our results, Watson et al. (2005) and Langlois et al. (2010) found that stereo‐BCs had more statistical power and were more cost‐efficient than stereo‐DOVs. Unlike UVCs, stereo‐DOVs require extensive postlaboratory analysis, and therefore are less cost‐efficient. However, this method may overcome some of the problems associated with UVCs, as it provides a permanent record, which can be reanalyzed and validated when required (Harvey, Fletcher, & Shortis, 2001). Furthermore, the larger transects used in their study might have contributed to a greater variability in the fish assemblage, as greater habitat heterogeneity would be recorded. A limitation of our study that might have averaged out variation of the fish assemblage, in the case of BCs, was the separation among adjacent BC units. A separation of >250 m between adjacent BCs is usually recommended, to minimize plume interferences, and therefore avoiding fishes moving from one unit to another. Hence, we might have incurred some sort of pseudo‐replication, reducing variation among replicated samples.

Our study highlights the need to implement complementary sampling techniques to monitor ecological change, at various dimensions of biodiversity. The results presented here might be useful for optimizing future monitoring programs. UVCs appear to be the most cost‐efficient technique, and it is recommended for local studies. However, for large‐scale studies, BCs appear to be a promising approach, as multiple systems are deployed simultaneously, improving the efficiency in the field. For example, while BCs might be more cost‐effective in the assessment of MPAs, where the protected area extends over large spatial scales, it might be less cost‐effective to assess environmental impacts that occur over smaller spatial scales (i.e., local impacts), as the individual replicates must be either separated by distances of >250 m or by time. Another advantage is that BCs can be deployed in a depth ranges that are inaccessible to divers, and are not limited by dive time and/or health and safety concerns. Future technological improvement in BC systems, for example, system autonomy, storage capacity, and sensor resolution, joined by the development of automated image analysis (e.g., www.Fish4Knowledge.eu, Phoenix, Boom, & Fisher, 2013) will be key for optimizing effort and might increase the cost‐effectiveness of this technique, especially for large‐scale studies.

## Conflict of Interest

None declared.
