# Supplementary material for: “How” and “what” matters: Sampling method affects biodiversity estimates of reef fishes
Source: Ecol Evol. 2017 May 30;7(13):4891–906. doi: 10.1002/ece3.2979 (PMC5496540; doi:10.1002/ece3.2979)
Supplement: Supplementary file 1 [file ECE3-7-4891-s001.docx]

Appendix 1. Fish traps (FT) used during this study.


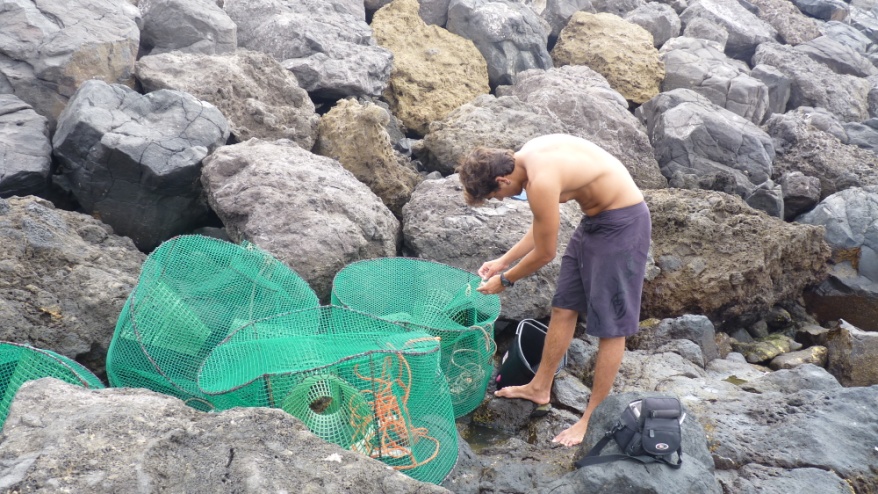


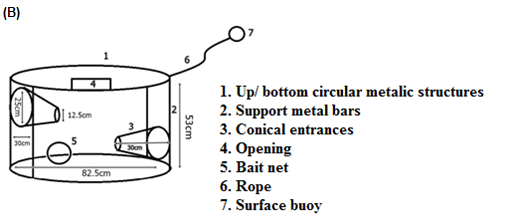


Appendix 2. Baited cameras (BC) used during this study.


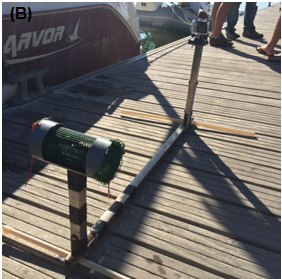


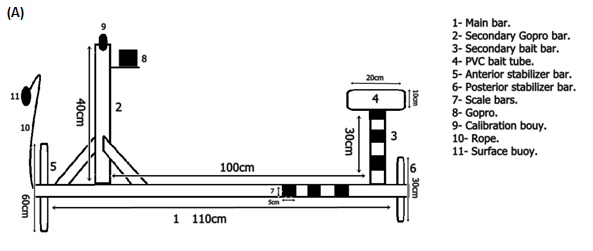


Appendix 3. Mean abundance (+SE) of species recorded by each sampling method: UVC (filled bars; ind 40 m^-2^), BC (grey bars; MaxN) and FT (unfilled bars; ind per trap) at Gran Canaria Island. Mean abundance is represented on a logarithmic scale. * = species of commercial relevance.


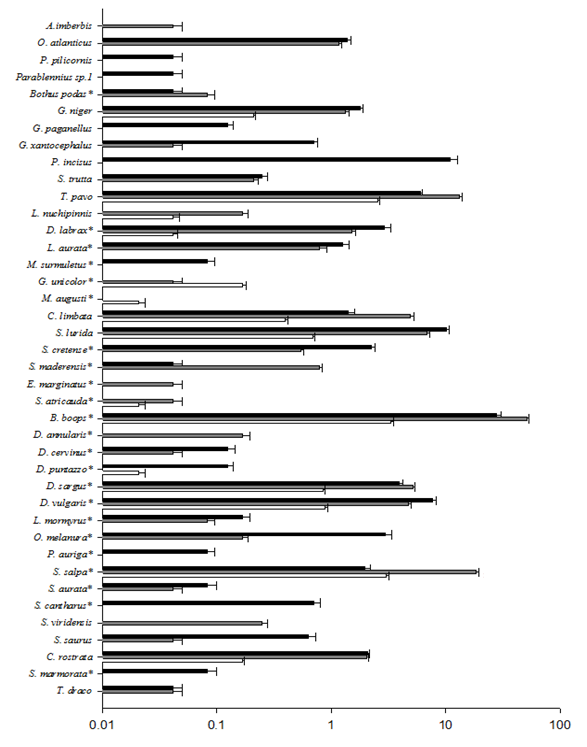


Appendix 4. Mean abundance (+SE) of species recorded by each sampling method: UVC (filled bars; ind 40 m^-2^), BC (grey bars; MaxN) and FT (unfilled bars; ind per trap) at southern Portugal data set. Mean abundance is represented on a logarithmic scale. * = species of commercial relevance.


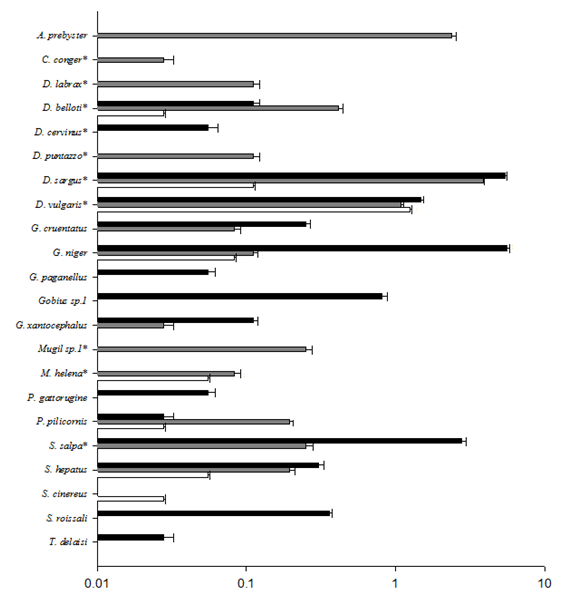


|  | Gran Canaria Island | | Southern Portugal | | |
| --- | --- | --- | --- | --- | --- |
|  | Inner | Outer | Inner | Middle | Outer |
| UVC | 0.89 | 0.81 | 1 | 0.84 | 0.92 |
| BC | 0.9 | 0.9 | 0.93 | 1 | 0.85 |
| FT | 1 | 0.58 | 0.82 | 0.77 | 0.73 |

Appendix 5. Achieved proportion of the estimated Chao 2 asymptotic richness for each method at each side within each region. N (sample size) = 12 for all methods, except for FT at Gran Canaria Island, N= 24.

Appendix 6. Effect size (f) and achieved power for the fixed effect ‘Side’ in the ANOVA model for each biodiversity index, according to underwater visual census (UVC), baited cameras (BC) and fish traps (FT). Significant values are highlighted in bold. * = cases when the achieve power was very low (i.e. < 0.4).

| Gran Canaria Island | UVC | | | BC | |  | FT | | |
| --- | --- | --- | --- | --- | --- | --- | --- | --- | --- |
| Index | Effect size (f) | Power (1-β) | P (Side) | Effect size (f) | Power (1-β) | P (Side) | Effect size (f) | Power (1-β) | P (Side) |
| H' | 0.32* | 0.32 | 0.13 | 0.38 | 0.43 | **0.05** | 0.21 | 0.3* | **0.01** |
| Δ* | 0.75 | 0.93 | **<0.001** | 0.76 | 0.94 | **<0.001** | 0.43 | 0.84 | **0.001** |
| Rao | 0.025 | 0.05 | 0.8 | 0.62 | 0.82 | **<0.001** | 0.28 | 0.49 | **0.01** |
| Southern Portugal | UVC | | | BC | |  | FT | | |
| Index | Effect size (f) | Power (1-β) | P (Side) | Effect size (f) | Power (1-β) | P (Side) | Effect size (f) | Power (1-β) | P (Side) |
| H' | 0.17 | 0.13* | 0.25 | 0.68 | 0.94 | **<0.001** | 0.22 | 0.18* | 0.3 |
| Δ* | 0.43 | 0.59 | **<0.001** | 0.29 | 0.3* | 0.07 | 0.23 | 0.2* | 0.3 |
| Rao | 0.38 | 0.49 | **<0.001** | 0.41 | 0.55 | **0.02** | 0.21 | 0.18* | 0.4 |
